# Polymorphisms of VEGF and VEGF receptors are associated with the occurrence of ovarian hyperstimulation syndrome (OHSS)—a retrospective case–control study

**DOI:** 10.1186/1757-2215-7-54

**Published:** 2014-05-13

**Authors:** Kazem Nouri, Peter Haslinger, Ladislaus Szabo, Michael Sator, Martin Schreiber, Christian Schneeberger, Detlef Pietrowski

**Affiliations:** 1 Department of Endocrinology and Reproductive Medicine, Medical University Vienna, Währinger Gürtel 18-20, 1090 Vienna, Austria; 2 Privatklinik Doebling, Fertility Center Doebling, Vienna, Austria

**Keywords:** ART, IVF-therapy, Vascular permeability, VEGF, OHSS

## Abstract

**Background:**

Ovarian hyperstimulation syndrome (OHSS) is the most serious complication of IVF/ICSI therapy. The pathophysiology and etiology of the disease is still not fully clarified.

**Methods:**

To assess whether polymorphisms of the VEGF/VEGF-receptor system contribute to the occurrence of ovarian hyperstimulation syndrome (OHSS), we performed a retrospective analysis of 116 OHSS patients, and 124 female controls. The following SNPs were genotyped: Rs2071559 (VEGFR2-604); rs2305948 (VEGFR2-1192); rs1870377 (VEGFR2-1719); rs2010963 (VEGF-405); and rs111458691 (VEGFR1-519). Odds ratios (ORs) were estimated with a 95% confidence interval (CI). Linkage disequilibrium (LD) analysis was performed in the three loci of the VEGFR2 gene.

**Result:**

We found an overrepresentation of the T allele of the VEGFR1-519 polymorphism in OHSS patients (P = 0.02, OR: 3.62, CI: 1.16 – 11.27). By genotype modeling, we found that polymorphism of VEGFR1-519 and VEGF-405 showed significant differences in patients and controls (p = 0.02, OR: 3.79 CI: 1.98 – 11.97 and p = 0.000005, OR: 0.29, CI: 0.17 – 0.50). LD analysis revealed significant linkage disequilibrium in VEGFR2.

**Conclusion:**

Polymorphisms in the VEGFR2 gene and in the VEGF gene are associated with the occurrence of OHSS. This strengthens the evidence for an important role of the VEGF/VEGF- receptor system in the occurrence of OHSS.

## Introduction

Iatrogenic ovarian hyperstimulation syndrome (OHSS) is the most serious complication of IVF/ICSI therapy. OHSS is characterized by ovarian enlargement and fluid shift from the intravascular to the extravascular compartment [1]. Although the etiology of OHSS is far from being fully understood, there are two frequently observed pathologies: (i) an increase in vascular permeability; and (ii) HCG is the triggering factor for this condition [2,3].

The mechanism by which HCG triggers vascular permeability in OHSS patients is still under investigation, but the vascular endothelial growth factor (VEGF) and its receptors seem to play a key role in this physiological process [4-7]. These observations are strengthened by the findings that the probability of developing OHSS correlates with the concentration of VEGF in serum [8-11] and that, in OHSS patients, the amount of VEGF in the follicular fluid is frequently higher than in individuals not affected by this complication [12]. Interestingly, both the clinical pregnancy rate and abortion rate in OHSS patients were significantly higher than that in patients without the syndrome [13,14]. These observations suggest that early pregnancy processes, i.e., implantation, trophoblast invasion, or placentation, are also affected in OHSS patients [15].

VEGF triggers its signalling via a set of three main receptors named VEGF receptor 1 to 3 (VEGFR1, VEGFR2, VEGFR3). However, only VEGFR1 and VEGFR2 are shown to be directly involved in VEGF binding [16,17].

Single nucleotide polymorphisms (SNPs) are described in the VEGF gene, as well as in VEGFR1 and VEGFR2. For some of these, a direct association with the transcriptional activity of the corresponding genes has been shown [18-20]. Polymorphisms in the VEGFR2 promoter region have been found to influence the transcription activity of VEGFR2, contributing to the development of coronary artery lesions (CAL) in patients with Kawasaki disease (KD) and various cancers [21-23].

In light of recent interest in the interplay between decreased serum concentration of soluble VEGF (sVEGF) receptors and an increased serum concentration of VEGF, our group hypothesized that the development of OHSS is based on the finding that the higher the concentration of VEGF and the lower the concentration of the sVEGF-receptors, the higher the probability of the occurrence of OHSS is [11].

## Materials and methods

In this case–control study, we included 116 women who suffered from severe OHSS during their IVF/ICSI therapy, and 124 healthy pregnant women who had delivered children in the department of OB/GYN at the General Hospital of Vienna (AKH). Institutional Review Board approval was obtained for the study at the MUW Ethikkommission (Reg. number 1398/2012).

We included all inpatients with the diagnosis of severe OHSS who required immediate hospitalization. The criteria for admission was met when at least two of the following factors were observed: 1) clinically relevant symptoms, such as diffuse abdominal discomfort, nausea, vomiting, dyspnea and tachypnea, subjective decreased urinary output; 2) electrolyte imbalance, leukocytosis >20,000/mm3, creatinine >1 mg/dL, and hematocrit >45%; and 3) sonographically detectable free fluid (ascites and/or pleural effusion), with a significant increase in ovarian diameter (>12 cm); and 3) clinical signs, such as oliguria <600 mL in 24 hours and hypotension.

Patients received stimulation with recombinant or human menopausal gonadotropins (hMG) after down-regulation via GnRHa (gonadotropin releasing hormone agonist) in case of a long protocol, or using Ganirelix (Orgalutran MSD Pharma) or Cetrorelix (Cetrotide MerckSerono) in case of an antagonist protocol.

All subjects received either 10,000 IU of hCG (Pregnyl, MSD Pharma), Choriogonadotropin alpha 250 μg (Ovitrell MerckSerono), or Triptorelin acetate 0.2 mg (Decapeptyl Ferring Austria) to trigger final oocyte maturation. Patients with any systemic disease were excluded from the study group and the control group. EDTA blood was obtained from all participants by peripheral antecubital vein puncture, and stored at-18°C until the analysis was performed.

### Standardized OHSS treatment

Once the patients were hospitalized with the diagnosis of OHSS III-IV according to Golan et al., they all received the same therapeutic protocol. Complete blood count with hematocrit, serum analysis of albumin, blood creatinine, blood urea, and creatinine clearance, liver function parameters, and coagulation tests on a daily basis were obtained in all patients. The amount of excreted urine, fluid balance, body weight, and abdominal circumference were carefully checked every 24 hours.

All patients were treated with 1000 ml lactated Ringer’s solution twice a day, with furosemide 20 mg (Lasix® 20 mg, Sanofi, Vienna, Austria) to maintain the diuresis. Plasma volume expansion with hydroxyethyl starch solution 6% (HAES-steril® 6% Infusionslösung, 250 ml KABI, Bd Homburg Germany), and/or human albumin 25% (Human Albumin® Octapharm, Langfeld, Germany) were given once daily in cases of hypoalbuminemia or right after paracentesis (>34 g/L).

If patients complained of pain or compromised pulmonary function, paracentesis was performed with a transvaginal approach.

Patients had to wear full-length venous support stockings. Prophylactic Enoxaparin therapy of 40 mg on a daily basis (Lovenox 40 mg, Sanofi-Aventis GmbH Vienna) was given to all patients during in-patient treatment. The prophylactic therapy with Cabergoline o.5 (Dostinex®, Pharmacia, Berlin, Germany) was given to all patients from January 2008, orally once a day within eight days after retrieval.

The duration of the hospitalization was dependent on two factors: i) resolution of the symptoms; and ii) normalization of the lab results. Patients were discharged from the hospital when their hematocrit, without receiving any infusions, remained under 40%.

### Genotyping

We determined the genotypes of five polymorphisms in three genes by polymerase chain reaction and restriction fragment polymorphism analysis: VEGFR1 (other name FLT1); VEGFR2 (other name KDR); and VEGF. We used the SNP rs111458691 for genotyping the VEGFR1-519 polymorphism, rs2071559, rs2305948, and rs1870377 for genotyping the VEGFR2-604, VEGFR2-1192 and VEGFR2-1719 polymorphisms, respectively, and rs2010963 for genotyping the VEGF-405 polymorphism.

To determine the genotypes, we amplified a genomic DNA fragment by polymerase chain reaction (PCR). PCR amplification was performed with 0.2 mM dNTP, 10 μM forward primer, 10 μM reverse primer, 1.5 mM MgCl2, and 1 U Taq polymerase (Sigma Qiagen, Hilden, Germany) in the recommended buffer. Cycling conditions were: 45 cycles in an Eppendorf Thermocycler at 94°C for 30 s, 60°C to 65°C for 30 s, and 72°C for 45 s after the initial denaturation step (94°C for 5 min). A seven-minute final extension step at 72°C was added. The total volume of the PCR reaction was 25 μl. The size of the PCR products, the enzymes used, and the resulting fragment length are given in Table 1. Fragments were visualized after electrophoresis on a 3%-4% agarose gel by staining with ethidium bromide. Pictures were taken with a Canon digital camera system (UVP Laboratory Products, USA).

**Table 1:** Primers, fragment length and enzymes used for RFLP analysis

**Gene/SNP**	**Primers (5′-3′)**	**Fragment lengths (bp)**	**Enzyme**	**RefSNP**
VEGFR2- 604	Fw: CAAACTTTCACTAGGGCTCTTCGT	290;174;116	B*sm*I	2071559
	Rev: AGCCACAAGGGAGAAGCGGATA			
VEGFR2-1192	Fw: TGAGGTTAAAAGTTCTGGTGTCCCTGTT	262;232;30	B*stZ17*I	2305948
	Rev: AATGTACAATCCTTGGTCACTCCGGGGTA			
VEGFR2-1719	Fw: CCTCCTGTATCCTGAATGAATCT	404;191;213	A*lu*I	1870377
	Rev: GCCTCACATATTATTGTACCATCC			
VEGF-405	Fw: ATTTATTTTTGCTTGCCATT	304;191;213	B*smF*1	2010963
	Rev: GTCTGTCTGTCTGTCCGTCA			
VEGFR1-519	Fw: GTGGCAACTTTGGGTTACCCA	665;520;145	N*sp*I	111458691
	Rev: CCTGACCCCTTCAGACTGTCC			

### Statistical analysis

Statistical analyses were performed using a chi-square test of association and a Fisher’s exact probability test at the Vassar Stats homepage ( http://vassarstats.net/). A value of P < 0.05 was defined as statistically significant. Odds ratios, confidence intervals, and linkage disequilibrium analyses were performed with R, an open-source language and environment for statistical computing [24]. To adjust the values for multiple comparisons, we used the Benjamini-Hochberg correction method, which controls for false discovery rate (FDR) [25].

## Results

Blood samples from 240 subjects (116 patients and 124 controls) were analyzed. An overrepresentation of the T allele of the VEGFR1-519 C > T polymorphism (rs111458691) was found in OHSS patients. The significant difference in the allele frequencies resulted in a 3.6-fold overrepresentation (p = 0.018), OR:3.62; CI: 1.16 – 11.27)). Although it did not reach a level of significance, a tendency toward overrepresentation of the A Allele of VEGFR2-1192 (rs2305948) in OHSS patients was also found (p = 0.06, OR:1.76; CI: 0.97 – 3.18). The allele frequencies of VEGFR2-604 (rs2071559), VEGFR2-1719 (rs1870377), and VEGFA-405 (rs2010963) were not statistically different (Table 2).

**Table 2:** Allele frequencies

		**OHSS (%)**	**Control (%)**	**P-value**^**a**^	**Chi**	**OR (CI)**
**VEGFR1-519**						
	C	219 (94.40)	244 (98.39)			
	T	13 (5.60)	4 (1.61)	0.018^*^	5.59	3.62 (1.16 - 11.27)
**VEGFR2-604**						
	T	107 (46.12)	125 (50.40)			
	C	125 (53.88)	123 (49.60)	0.35	0.88	1.19 (0.83- 1.7)
**VEGFR2-1192**						
	G	201 (86.64)	228 (91.94)			
	A	31 (13.36)	20 (8.06)	0.06	5.59	1.76 (0.97 - 3.18)
**VEGFR2-1719**						
	T	55 (23.71)	60 (24.19)			
	A	177 (67.29)	188 (75.81)	0.88	0.02	0.97 (0.64 - 1.48)
**VEGF-405**						
	G	91 (39.22)	108 (43.55)			
	A	141 (60.78)	140 (5645)	0.34	0.92	0.84 (0.58 - 1.20)

^a^Chi square test was used, with p < 0.05 considered significant and marked with an asterisk. To adjust the values for multiple comparisons, we used the Benjamini-Hochberg (BH) correction method, which controls false discovery rate (FDR).

We calculated different genotype models and considered the model with the smallest probability value as the best–fitting model for the respective genotype. Using an overdominant genotype model, we found that VEGF-405 polymorphism is significantly associated with OHSS (p = 0.000005; OR:0.29; CI: (0.17 – 0.50), and we found a significant association of VEGFR1-519 SNP with OHSS (p = 0.014; OR: 3.79; CI: 1.2 – 11.97), using a co-dominant genotype model.

No significant difference in genotype distribution models was found for VEGFR2-604, VEGFR2-1192, and VEGFR2-1719 (Table 3).

**Table 3:** Genotypes

	**Control**	**%**	**Patient**	**%**	**OR**	**Lower**	**Upper**	**p-value**^**a**^
**VEGFR1-519**								
(Co-dominant)								
C/C	120	96,8	103	88,8	1,00	-	-	0,01*
C/T	4	3,2	13	11,2	3,79	1,20	11,97	
T/T	0	-	0	-	-	-	-	
**VEGFR2-604**								
(Co-dominant)								
C/C	22	17,7	28	24,1	1	-	-	0,47
C/T	79	63,7	69	59,5	0,69	0,36	1,31	
T/T	23	18,5	19	16,4	0,65	0,28	1,48	
**VEGFR2-1192**								
(Co-dominant)								
G/G	106	85,5	90	77,6	1	-	-	0,22
G/A	16	12,9	21	18,1	1,55	0,76	3,14	
A/A	2	1,6	5	4,3	2,94	0,56	15,54	
**VEGFR2-1719**								
(Co-dominant)								
A/A	69	55,6	66	56,9	1	-	-	0,97
A/T	50	40,3	45	38,8	0,94	0,56	1,59	
T/T	5	4,0	5	4,3	1,05	0,29	3,78	
**VEGF-405**								
(Recessive)								
A/A-A/G	116	93,5	97	83,6	1	-	-	0,01*
G/G	8	6,5	19	16,4	2,84	1,19	6,77	

^a^Chi square test was used, with p < 0.05 considered significant and marked with an asterisk.

The data from the three SNPs of VEGFR2 were tested for redundancy by linkage disequilibrium (LD) analyses. This analysis showed a significant LD for SNP VEGFR2-604 vs. VEGFR2-1192 (D’ = 0,44; r = -0.15; p = 0.001) and for VEGFR2-1192 vs. VEGFR2-1719 (D’ = 0.31; r = 0.19; p = 0.00003). However, this did not reveal a significant correlation between VEGFR2-604 and VEGFR2-1719.

Since we followed a standardized therapy for all our patients, we defined the day of discharge from the hospital as the day when the symptoms of OHSS were resolved. In Table 4, we examined the correlation between the duration of the hospitalization between the patients with and without the investigated polymorphisms. Therefore, we grouped patients into two groups with regard to their stay in our clinic (Group 1: one to nine days; group 2: 10 to 28 days; mean of all samples: 10.03 days). This subgroup analysis showed no statistically significant difference between patients who stayed longer than nine days in the hospital and patients who stayed between one and nine days in our clinic for every analyzed SNP.

**Table 4:** Subgroup analysis

**Allele frequencies in accordance to hospital stay**						
		**OHSS-S (%)**	**OHSS-L (%)**	**P-value**	**Chi**	**OR (CI)**
**Mean (Range)**		6.58 (2–9)	13.36 (10 – 28)			
**VEGFR1-519**	C	107 (93.9%)	112 (94.9%)			
	T	7 (6.1%)	6 (5.1%)	0.78	0.73	1.22 (0.39 - 3.75)
**VEGFR2-604**	T	56 (49.1%)	51 (43.2%)			
	C	58 (51.9%)	67 (56.8%)	0.79	0.78	0.93 (0.55 - 1.57)
**VEGFR2-1192**	G	99 (86.8%)	102 (86.4%)			
	A	15 (13.2%)	16 (13.6%)	1.0	0.92	0.97 (0.45 - 2.1)
**VEGFr2-1719**	T	25 (21.9%)	30 (25.4%)			
	A	89 (78.1%)	88 (74.6%)	0.54	0.53	1.22 (0.66 - 2.23)
**VEGF-405**	G	42 (36.8%)	49 (41.5%)			
	A	72 (63.2%)	69 (58.5%)	0.50	0.47	1.22 (0.72 - 2.06)

Legend: Allel frequencies and p values are given for association of SNPs and duration time in hospital. OHSS-S: Duration in hospital 1–9 days, OHSS-L: Duration in hospital 10–28 days.

## Discussion

In this study, we investigated the association of five polymorphisms in three genes of the VEGF/VEGF receptor system and the occurrence of OHSS in 116 patients who suffered from OHSS and 124 healthy pregnant women. We analyzed a more general group of patients as controls, namely, healthy pregnant women, because this group represents a cross-sectional control compared to IVF patients, and is not selected by various fertility-related pathologies that are characteristic of IVF patients.

We found a significant association of the VEGF405 polymorphism (rs2010963) and the VEGFR1-519 polymorphism (rs111458691) with the occurrence of OHSS. However, the absolute difference of 5.6*%* and 1.6*%* in VEGFR1-519 T SNP for patients with OHSS shows that this SNP seems to be a rather rare allele.

The finding of a statistically significant association of the VEGF-405 SNP and OHSS development confirms and extends previous findings by Hanevik et al., who demonstrated an association of this SNP and OHSS in a study group of 53 Norwegian patients suffering from OHSS and a control group that performed IVF therapy but did not suffer from this complication [26]. Our collective comprised 116 OHSS patients with a presumably different Caucasian ethnicity than in Hanevik’s study, and were compared to healthy pregnant women. Although our findings might strengthen the validity of a correlation between OHSS and the VEGF-405 genotype, these findings also open the possibility of a correlation between other fertility-related pathologies (e.g., PCOS, endometriosis) with this SNP that require IVF therapy. In both cases, this genotype variation might be of biological significance because it has been shown by other authors that this genotype contributes to VEGF serum levels [20] and that VEGF serum levels are associated with OHSS, PCOS, and endometriosis [27-29]. In addition, Watson et al. identified a predicted myeloid zinc finger binding site (MZF1) at polymorphism VEGF-405 that opens the possibility that variations at this site directly affect VEGF gene expression through a different binding efficiency of zinc finger transcription factors at its recognition site [18].

To the best of our knowledge, we show, for the first time, an association between the VEGF-receptor polymorphism VEGFR1-519 (rs111458691) and the occurrence of OHSS.

Previously, polymorphisms in the VEGF receptors were shown to be associated with some common cancers, stroke, systemic lupus erythematosus, and recurrent pregnancy loss [23,30-33]. Moreover, in colon and rectal cancer, it was suggested that the VEGF receptors have a greater influence on cancer risk than VEGF, and it is thought that this effect might be caused by an interaction of the VEGF receptors with inflammatory signals [23].

An analysis of the VEGFR1-519 SNP by Menendez et al. demonstrated that the T-allele generates a p53 response element, placing the VEGF-VEGF receptor system directly in the p53 stress response transcriptional network [34]. In our study, we found an overrepresentation of the T-allele in OHSS patients. This opens the intriguing possibility that p53-mediated stress signalling indirectly contributes to the pathology of OHSS, and led us to suggest a role for p53 in the pathology of OHSS. This idea is strengthened by the recent findings of Boudjenah et al., showing that p53 polymorphisms could influence the ovarian response to rFSH stimulation for patients undergoing an intracytoplasmic sperm injection program [35]. In a subgroup analysis of the OHSS patient group, we correlated the hospital stay with the various SNPs. This analysis revealed no significant differences in allele frequencies between patients who have a short stay in the hospital (one-to-nine days) and patients who required a longer habitation (10–28 days). This finding suggests that the severity of OHSS based on hospital stay is not associated with any of the analyzed polymorphisms.

Our study has some limitations. This is a retrospective study and some data, such as full infertility anamnesis, possible medication effects, and the exact IVF protocol—including E2 concentration on the day of retrieval—are missing because patients were included from other clinics after their OHSS symptoms required medical care in a specialized center. In addition, we found that the polymorphism VEGF-405 is out of the Hardy Weinberg equilibrium (HWE). Based on the small amount of possible further information available about our study population, a selection bias could not be excluded.

## Conclusion

In the present study, we found a significant association of the VEGF405 polymorphism (rs2010963) and the VEGFR1-519 polymorphism (rs111458691) with the occurrence of OHSS. The identification of a context between OHSS and specific gene variants of the VEGF/VEGF receptor system involved in vascular permeability enabled further insights concerning the pathophysiology and etiology of OHSS, and may contribute to a genetic characterization of susceptible women before beginning an ART program. Although the present study may provide informative and supportive data relating to the clinical significance of OHSS and its relationship to the VEGF/VEGF-receptor system, future larger studies should be performed to fully explore this putative gene–epidemiology interaction.

## Competing interests

The authors declare no competing interests.

None of the authors have any relevant financial, personal, political, or religious interest linked to the subject of this article.

## Authors’ contributions

KN contributed to patient recruiting, interpretation of data, and manuscript writing and editing; PH and LS performed data collection and data mining, M Sch and CS contributed to data analysis, interpretation of data, and manuscript editing. MS contributed to patient recruiting and correcting the manuscript. DP analyzed and interpreted the data, contributed to manuscript writing and editing, and supervised the project. All authors read and approved the final manuscript.
